# Peritoneal Dialysis-Associated Peritonitis in 2 Patients with Bartter Syndrome and End-Stage Kidney Disease: A Case Report

**DOI:** 10.1159/kbr/adjag004

**Published:** 2026-08-19

**Authors:** Caterina Vitagliano, Giulia Florio, Giovanni Piscopo, Francesca Silvestri, Roberto Russo, Ida Molfino, Anna Iervolino, Flavia Maria Longo, Roberta Ranieri, Miriam Zacchia, Alessandra F. Perna, Loreto Gesualdo, Francesco Trepiccione

**Affiliations:** a Department of Medical Translational Sciences, University of Campania “Luigi Vanvitelli”, Naples, Italy; b Nephrology, Dialysis and Transplantation Unit, Department of Emergency and Organ Transplantation, University of Bari Aldo Moro, Bari, Italy; c Biogem, Institute of Molecular Biology and Genetics, Ariano Irpino, Italy

**Keywords:** Bartter syndrome, Peritoneal dialysis, End-stage kidney disease, Peritonitis

## Abstract

**Introduction:**

Bartter syndrome (BS) is a rare inherited salt-wasting disorder affecting the thick ascending limb of the loop of Henle. It is characterized by hypokalemic metabolic alkalosis, polyuria, and secondary normotensive hyperaldosteronism. A minority of patients may progress to end-stage kidney disease while maintaining polyuria and normokalemia. Peritoneal dialysis (PD) may represent a suitable option for these patients when kidney transplantation is not available. However, due to its rarity, there are no data on PD-related outcomes.

**Methods:**

Here, we report 2 cases of PD treatment in BS from two Italian referral centers.

**Results:**

In both cases, PD was discontinued early because of peritonitis.

**Conclusion:**

Although no causal relationship can be established, these observations highlight the need for heightened vigilance regarding infectious complications in this population.

## Introduction

Bartter syndrome (BS) is an inherited salt-losing tubulopathy characterized by hypokalemia, hypochloremic metabolic alkalosis, polyuria, and hyperaldosteronism with normo/hypotension, first described by Bartter in 1962 [1]. Due to different molecular TAL mechanisms, five types of BS have been identified [2]. BS type I is caused by loss-of-function mutations in *SLC12A1,* encoding the apical NKCC2 cotransporter, whereas mutations in *KCNJ1*, encoding the luminal potassium channel ROMK, lead to BS type II. Variants in the *CLCNKB* gene, encoding a basolateral TAL chloride channel, cause BS type III, while simultaneous mutations in *CLCNKB* and *CLCNKA* or in the barttin subunit are responsible for BS types IVa and IVb, respectively. In addition, mutations in *MAGE-D2* have been associated with transient BS. Recently, some studies have demonstrated pendrin dysfunction may also be implicated in blood pressure modulation [3], leading to a salt-losing tubulopathy without the classical Pendred syndrome [4].

As part of disease management, particularly in antenatal presentation (BS type I and type II), COX-1 and COX-2 inhibitors play an important role. Indomethacin, ibuprofen, and celecoxib are the most frequently used drugs. Although these therapies are usually initiated in childhood, there is discontinuation due to perceived lack of efficacy at school age, although there is no clear guideline on it.

BS has also been associated with progression toward end-stage kidney disease (ESKD) [2], although the burden and determinants of this progression remain incompletely understood. Among the most likely ones, chronic hypokalemia, frequent dehydration, higher copeptin levels [5] and chronic use of NSAIDs seem to play an additive role. Several reports have also described glomerular involvement in BS, frequently associated with a focal segmental glomerulosclerosis pattern [6, 7].

The optimal kidney replacement strategy for BS patients remains unclear. Since polyuria and hypokalemia can also persist in terminal stages, peritoneal dialysis (PD) may represent a suitable option as a bridge to organ transplantation. In addition, it offers continuous and gradual removal of fluid and solutes, resulting in more stable hemodynamics and better preservation of residual kidney function than hemodialysis. However, due to the rarity of this condition, data on its performance are currently lacking. Indeed, since they live their entire life in a hyperreninemic hyperaldosteronism state, it is not clear whether this per se affects the peritoneal membrane filtration status. Moreover, previous studies have suggested immune dysregulation in salt-losing states [8], including T-cell dysfunction with impaired IL17 responses, and it is not clear whether this can impact the development of peritonitis. Here, we report data from 2 cases of BS patients at CKD stage V on PD that may provide some preliminary insights into these unresolved questions.

## Case Report 1

A 36-year-old woman with BS type I was referred to our center because of worsening kidney function. Due to severe polyhydramnios, she was born prematurely at 28 weeks of gestation to non-consanguineous parents. She had been receiving lifelong KCl supplementation and indomethacin (50 mg/day) from 3 to 22 y/o.

At age 34, she developed primary hyperparathyroidism secondary to right inferior parathyroid adenoma and subsequently underwent thyroidectomy and partial parathyroidectomy. At 28 y/o, she developed nephrotic-range proteinuria. Kidney biopsy was not performed, and progression to ESKD occurred at 37 y/o. At that time, a Tenckhoff peritoneal catheter was inserted. Her physical examination showed no peripheral edema, low-to-normal blood pressure, and preserved residual urine output of approximately 1.5 L/day. She started with an incremental CAPD regimen consisting of two daily exchanges with 2 L each. After 2 weeks, asymptomatic hypocalcemia occurred (sCa 6.6 mg/dL, albumin 3.9 mg/dL, PTH 122 pg/mL, r.r. 4.6–58.1 pg/mL). Calcium mass transfer (CMT) across the peritoneal membrane was positive both with dialysate containing [Ca^2+^] 1.25 mm and 1.75 mm (CMT 0.4 and 1.8, respectively), suggesting preserved calcium transfer through the peritoneal membrane. Indeed, dialysate calcium concentration of 1.75 mmol/L was prescribed to improve serum calcium levels. The Peritoneal Equilibration Test classified the patient as a fast transporter (D/P creat.: 0.89, ΔNa: <5 mmol/L, and UF: >400 mL).

The patient experienced an unusually high rate of peritoneal infections compared to the center experience (0.34 peritonitis episodes/patient-year). Indeed, the first episode occurred 6 months after PD initiation. No pathogen was identified, and peritonitis resolved with empirical antibiotic therapy using ceftazidime and vancomycin. Two relapsing Tenckhoff catheter infections due to *Staphylococcus haemolyticus* occurred, requiring catheter replacement. The patient underwent kidney transplantation a few months later.

## Case Report 2

A male patient was diagnosed with BS at the age of 1 year because of failure to thrive and recurrent vomiting. He was born at term and subsequently diagnosed with BS type III. He received KCl supplementation and indomethacin treatment until adulthood. He was lost to follow-up during adulthood until he was reevaluated at the age of 28 y/o when CKD V was diagnosed. After 2 years of conservative therapy, at the age of 30 y/o, he started PD with a full-dose CAPD regimen consisting of four daily exchanges of 2 L of 1.36% glucose dialysate. After 1 month, treatment was switched to APD with 10 L of 1.36% glucose dialysate overnight. After 6 months of treatment, he developed an *Achromobacter xylosoxidans* multiple drug-resistant-mediated peritonitis requiring Tenckhoff catheter removal and transfer to hemodialysis.

## Discussion

Extensive data on long-term kidney outcomes in patients affected by BS are lacking. However, nephrotic range proteinuria has been reported [6] along with biopsy findings of glomerular and tubulointerstitial lesions often associated with a pathological pattern of FSGS [9]. Both patients in our report developed ESKD in the setting of glomerulopathy. Although progression to ESKD in BS is uncommon, BS I and BS IV appear to carry an increased risk compared to BS II and BS III [9, 10]. Chronic hypokalemia, recurrent dehydration with hypovolemia, and prolonged NSAID exposures have also been proposed as additional contributing factors, and their role in our cases cannot be excluded.

Following PD initiation, patient 1 developed hypocalcemia requiring oral calcium supplementation, active vitamin D and 1.75 mm [Ca^2+^] dialysate solution. Because this patient had previously undergone partial parathyroidectomy, a relatively frequent condition in BS [11], we could not exclude the contribution of this factor to the development of hypocalcemia despite residual PTH 122 pg/mL. Interestingly, a similar finding has previously been reported in a patient with Gitelman syndrome without a prior history of parathyroidectomy [12].

The most remarkable observation in our cases was the early occurrence of PD-related infectious complications. This appeared unusual considering the relatively low peritonitis rates reported by the two centers (0.344 and 0.255 episodes/patient-year for centers 1 and 2, respectively, according to the Italian Registry of Peritoneal Dialysis). Furthermore, low education level and cognitive impairment, well-known risk factors for peritonitis development [13], were not present in our patients (Table 1)*.*

**Table 1.** Clinical characteristics of 2 patients with BS who progressed to ESKD

​	Patient 1	Patient 2
BS	Type 1	Type III
Genetic	SLC12A1: c.903delC	CLCNKB: c.[1313G>A] and CLCNKA–CLCNKB deletion^a^
Diagnosis of CKD	Second decade of life	Second decade of life
Nephrocalcinosis	NO	NO
Education level	ISCED 7	ISCED 3
Age during ESKD, years	37	30
Pre-PD serum Ca^2+^, mg/dL	7.3	8.4
PD vintage, years	1	0.5
2-Week PD Ca^2+^, mg/dL	6.6	8.5
Infections, *n*	3	1
Exit-site infections	NO	NO
Other infections	NO	UTI

^a^ CLCNKB (NM_000085) c.1313G>A, p.(Arg438His), maternally inherited, in compound heterozygosity with a contiguous CLCNKA–CLCNKB deletion (arr[GRCh37] chr1:16351556–16387631). SLC12A1 (NM_000338) c.903delC; p.(Arg302Glyfs*3).

CKD, chronic kidney disease; ESKD, end-stage kidney disease; PD, peritoneal dialysis; ISCED, International Standard Classification of Education; UTI, urinary tract infection.

Previous studies suggested that salt-losing tubulopathies may be associated with altered immune responses, including impaired IL-17 signaling and an increased prevalence of bacterial and fungal infections, allergic manifestations, and autoimmune phenomena [8]. In addition, uremia, per se, is associated with immune dysfunction and downregulation of T-cell responses [14], potentially representing an additional contributing factor in our patients.

While causality cannot be inferred from 2 cases, the occurrence of PD-related infectious complications in both patients adds to the limited clinical experience with PD in BS. Given the rarity of BS progressing to ESKD, we believe these observations may deserve attention and warrant further investigation in larger cohorts. These findings could suggest the need for increased clinical awareness regarding infectious complications as well as perhaps a more careful surveillance strategy during early phases of PD treatment.

The CARE checklist is findable in online supplementary material (for all online suppl. material, see https://doi.org/10.1159/kbr/adjag004).

## Acknowledgments

The two clinical centers involved in this study are part of the ERKNET consortium. This study has been accomplished according to the mission of the ERKNET consortium to improve the knowledge on rare kidney disease.

## Statement of Ethics

This study was performed in accordance with the Declaration of Helsinki. Ethical approval was not received for this human study because, in accordance with local and national guidelines, it was not required. All adult participants provided written informed consent to participate in this study. Written informed consent was obtained from the individuals for publication of the details of their medical case and any accompanying images.

## Conflict of Interest Statement

Miriam Zacchia, Alessandra Perna, and Francesco Trepiccione were members of the journal’s Editorial Board at the time of submission. The other authors have no conflicts of interest to declare.

## Funding Sources

This study was not supported by any sponsor or funder.

## Author Contributions

F.T., C.V., M.Z., and G.P. designed the study and drafted the manuscript; G.F., R.R., I.M., A.I., R.Ra., F.S., and F.M.L. contributed to data collection and analysis; A.F.P. and L.G. revised the manuscript and conceptualization.

## Data Availability

All data generated or analyzed are in this article and its online supplementary material. Further inquiries can be directed to the corresponding author.
